# Removal of jammed loop cutter and residual snare wire, after polypectomy with a detachable snare

**DOI:** 10.1055/a-2439-3733

**Published:** 2024-11-08

**Authors:** Kei Nakazawa, Yuki Hirata, Ryoji Koshiba, Kazuki Kakimoto, Takako Miyazaki, Shiro Nakamura, Hiroki Nishikawa

**Affiliations:** 1 13010 2nd Department of Internal Medicine, Osaka Medical and Pharmaceutical University, Takatsuki, Japan

Detachable snares, which are tightened around the polyp base during the resection of pedunculated polyps, are very useful for preventing bleeding during polypectomy 1 2 3 . However, unexpected situations are occasionally encountered when using detachable snares. Here, we present a case in which, after ligation with a detachable snare, the excess snare wire became wedged through the loop cutter, jamming it so that its cutting mechanism could not be opened or closed.

A 64-year-old man with a positive fecal occult blood test was referred to our hospital for colonoscopy. This revealed a pedunculated polyp, approximately 20 mm in size, in the sigmoid colon ( Fig. 1 ). To prevent bleeding, ligation was done using a detachable snare at the base of the polyp ( Fig. 2 ). The polyp was then removed. However, when we tried to cut the residual snare wire, it got wedged in the loop cutter. This jammed the loop cutter and it could neither be opened or closed. Ultimately, the sheath of the detachable snare was excised near the endoscopic instrument channel using nippers, and the scope was removed from the colon. The scope was subsequently reinserted and the snare wire was successfully resected using another loop cutter. The jammed loop cutter was removed from the colon along the sheath ( Fig. 3 , Video 1 ).

**Fig. 1:**
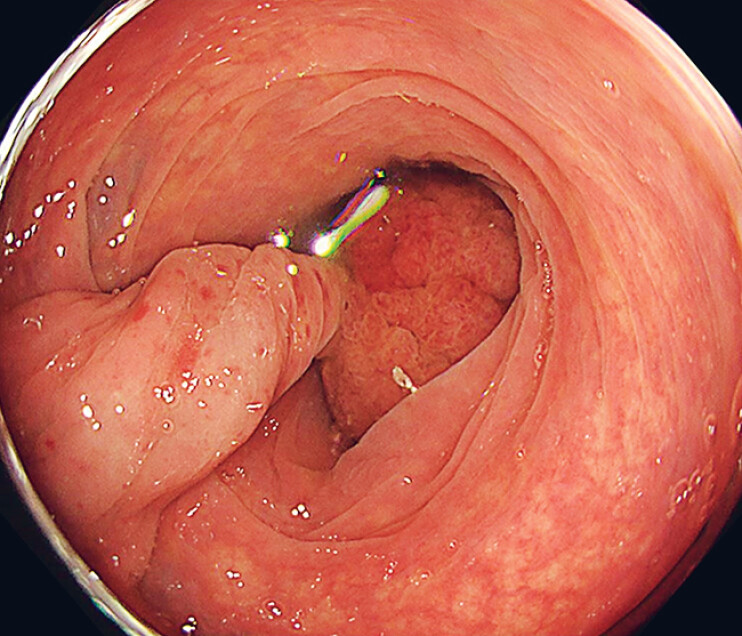
A pedunculated polyp of about 20 mm was identified in the sigmoid colon, with a twisted and pulsating stem that suggested the presence of an artery.

**Fig. 2:**
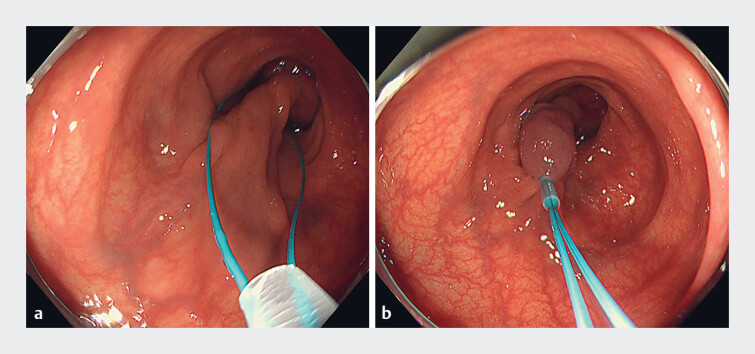
**a** Detachable snare placed around the base of the polyp. **b** Ligation achieved cessation of the blood flow.

**Fig. 3:**
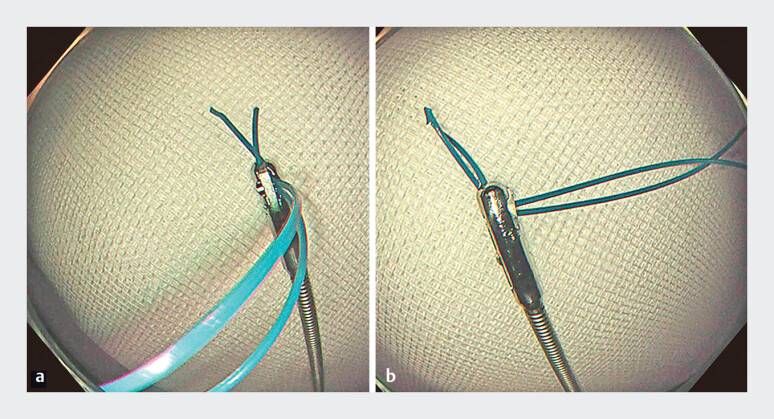
**a, b** The jammed loop cutter cannot be opened or closed because the residual snare wires have become lodged in the cutter.

Dealing with loop cutter jammed by residual snare wire, after polypectomy using a detachable snare for prevention of bleeding.Video 1

If a cut is attempted tangentially to the excess snare wire, the wire can become wedged in a gap at the base of the cutting mechanism. This jams the loop cutter so that it cannot be opened or closed. Therefore, it is important to perform cutting in the perpendicular direction ( Fig. 4 ). To the best of our knowledge, this is the first video report in which a jammed loop cutter could not be opened or closed, making this a valuable case.

**Fig. 4:**
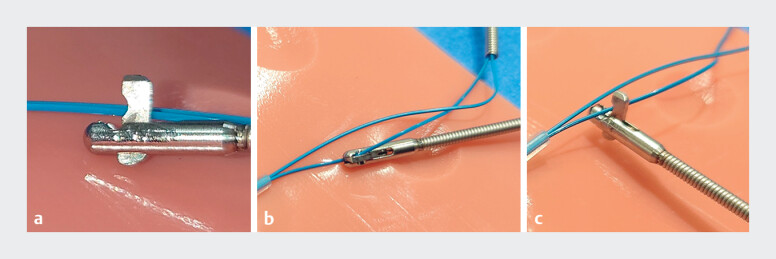
Model of the complication. **a** A gap can be observed at the base of the loop cutter. **b** Cutting in a tangential direction wedges the snare wire and jams the cutting mechanism. **c** Cutting should be done perpendicular to the wire.

Endoscopy_UCTN_Code_CPL_1AJ_2AD_3AB

## Notes

Endoscopy E-Videos https://eref.thieme.de/e-videos
*E-Videos* is an open access online section of the journal *Endoscopy* , reporting on interesting cases and new techniques in gastroenterological endoscopy. All papers include a high-quality video and are published with a Creative Commons CC-BY license. Endoscopy E-Videos qualify for HINARI discounts and waivers and eligibility is automatically checked during the submission process. We grant 100% waivers to articles whose corresponding authors are based in Group A countries and 50% waivers to those who are based in Group B countries as classified by Research4Life (see: https://www.research4life.org/access/eligibility/ ). This section has its own submission website at https://mc.manuscriptcentral.com/e-videos .
